# Meta-analysis of controlled trials testing horticultural therapy for the improvement of cognitive function

**DOI:** 10.1038/s41598-020-71621-7

**Published:** 2020-09-03

**Authors:** Hung-Ming Tu, Pei-Yu Chiu

**Affiliations:** Department of Horticulture, National Chung Hsing University, Taichung, 40227 Taiwan

**Keywords:** Health care, Quality of life, Therapeutics

## Abstract

Improving cognitive function is one of the most challenging global issues in cognitive impairment population. Horticultural therapy involves the expertise of a horticultural therapist who establishes a treatment plan for horticultural activities that aim to achieve cognitive changes, and thereby improve health-related quality of life. However, more convincing evidence demonstrating the effect of horticultural therapy on cognitive function is essential. The purpose of this study was to conduct a meta-analysis of controlled trials testing the effect of horticultural therapy on cognitive function and the findings indicate that horticultural therapy programs significantly improved cognitive function. The effect size of the horticultural therapy program was large. Findings of this meta-analysis have important implications for practice and policies. Contemporary healthcare systems should consider horticultural therapy as an important intervention for improving patients’ cognitive function. Governments and policy-makers should consider horticultural therapy as an important tool to prevent the decline of cognitive function in cognitive impairment population.

## Introduction

Population aging has led to serious healthcare problems in many countries. The decline of cognitive impairment is one of the characteristics of the aging population. Cognitive impairment affects social support^1^, quality of life^2^, and health^3,4^ and increases the risk of dementia^5,6^. When cognitive impairment progresses to dementia, the resultant potential economic and social burdens can be substantial^7,8^, especially on caregivers^9^. The promotion of cognitive function or prevention of cognitive impairment has become one of the most challenging issues in the world. Horticultural therapy involves the expertise of a horticultural therapist who establishes a treatment plan involving horticultural activities that aims to achieve specific goals^10^ which results in psychological, physiological, and cognitive changes that improve health-related quality of life^11–13^ and biological changes that prevent chronic diseases^14^.

Several theories explained the impact of horticultural therapy on cognitive function. The attention restoration theory states that viewing natural settings and natural plants may restore direct attentional capacity from fatigue and induce the feeling of being-away from stress^15,16^. Horticultural therapy establishes a sense of control, empowerment, and cooperation to satisfy the requirement of safety and stabilization from cognitive overburden status according to trauma recovery concept^17^. Horticultural therapy provides reminiscence benefits to promote memory and cognitive skills through olfactory sense and gustatory sense^18^. Based on the above theories, horticultural therapy may improve attention, a sense of control, memory and cognitive skills.

In many countries, for example Taiwan, improvement of cognitive function through horticultural therapy is not considered within mainstream medical and healthcare systems due to a lack of supporting scientific evidence. There are some trials in the literature that support the positive effects of horticultural therapy on cognitive function; however these studies do not have control groups^18–21^. Higher level evidence is lacking. There are also some randomized controlled trials available, but of these, some are compromised by ambiguous methodology e.g., randomization and blinding methods, low reporting quality and heterogeneity of the results^22^. High-quality randomized controlled trials are scarce in horticultural therapy because they are the most challenging to design and implement. Evidence from available non-randomized, controlled trials have shown that horticultural therapy programs did not affect cognitive function^17,23,24^. One possible reason is that the sample sizes are small, and therefore statistically significant treatment effects are not able to be detected^25^.

Meta-analysis is a useful method to address research challenges such as small sample sizes and lowered statistical power. It computes a summary effect of individual studies through the process of search strategy, data extraction, quality assessment, and data analysis^25^. Surprisingly, only one study, Soga et al.^26^, used meta-analysis to analyze the comprehensive health benefits of gardening and horticultural therapy. However, the study of Soga et al.^26^ included non-horticultural therapy, did not report each study’s quality assessment, and only included studies with more than 11 participants. There is an urgent need to focus analysis on studies that determine the effect between horticultural therapy and cognitive function alone. The meta-analysis of controlled trials will likely provide a higher level of evidence. Therefore, this meta-analysis of controlled trials aimed to investigate the effect of horticultural therapy on cognitive function. In the level of evidence, the controlled trials without randomization stand at a higher level than case–control, cohort studies, and descriptive studies although meta-analysis of all randomized controlled trials have the highest level of evidence^27^. However, few horticultural therapy studies are randomized controlled trials. Therefore, this study focused on not only randomized controlled trials but also well-designed controlled trials without randomization. The hypothesis is that horticultural therapy has a positive effect on cognitive function.

## Results

### Characteristics of the included studies

**Figure 1:**
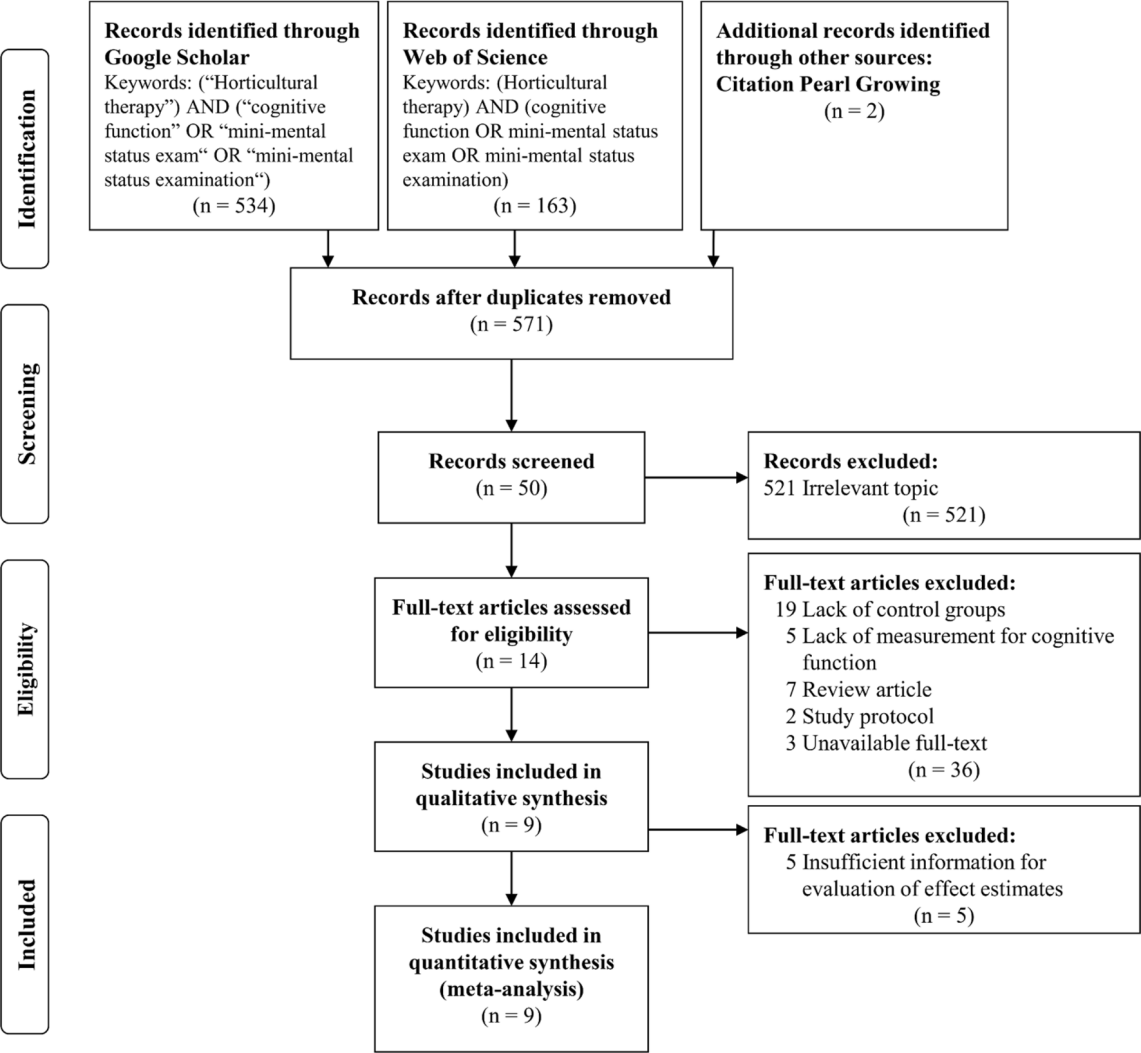
Preferred reporting items for systematic reviews and meta-analyses (PRISMA)^43,44^ flow diagram for search, selection, and identification process of this study.

A total of 571 records were searched and identified after duplicates were removed (Fig. 1). After 521 irrelevant topics were removed, 50 studies on horticultural therapy were screened. Of these, 36 full-text articles were excluded (including 19 without control groups, 5 that did not measure cognitive function, 7 review articles, 2 study protocols and 3 abstracts, resulting in 14 full-text articles that met inclusion eligibility. The final analyzed dataset included 9 articles^17,23,24,28–33^, after qualitative synthesis and feasibility evaluation of effect estimates which excluded 5 articles due to insufficient information for the evaluation of effect estimates. Krippendorff’s alpha was 0.95, indicating good reliability.

**Figure 2:**
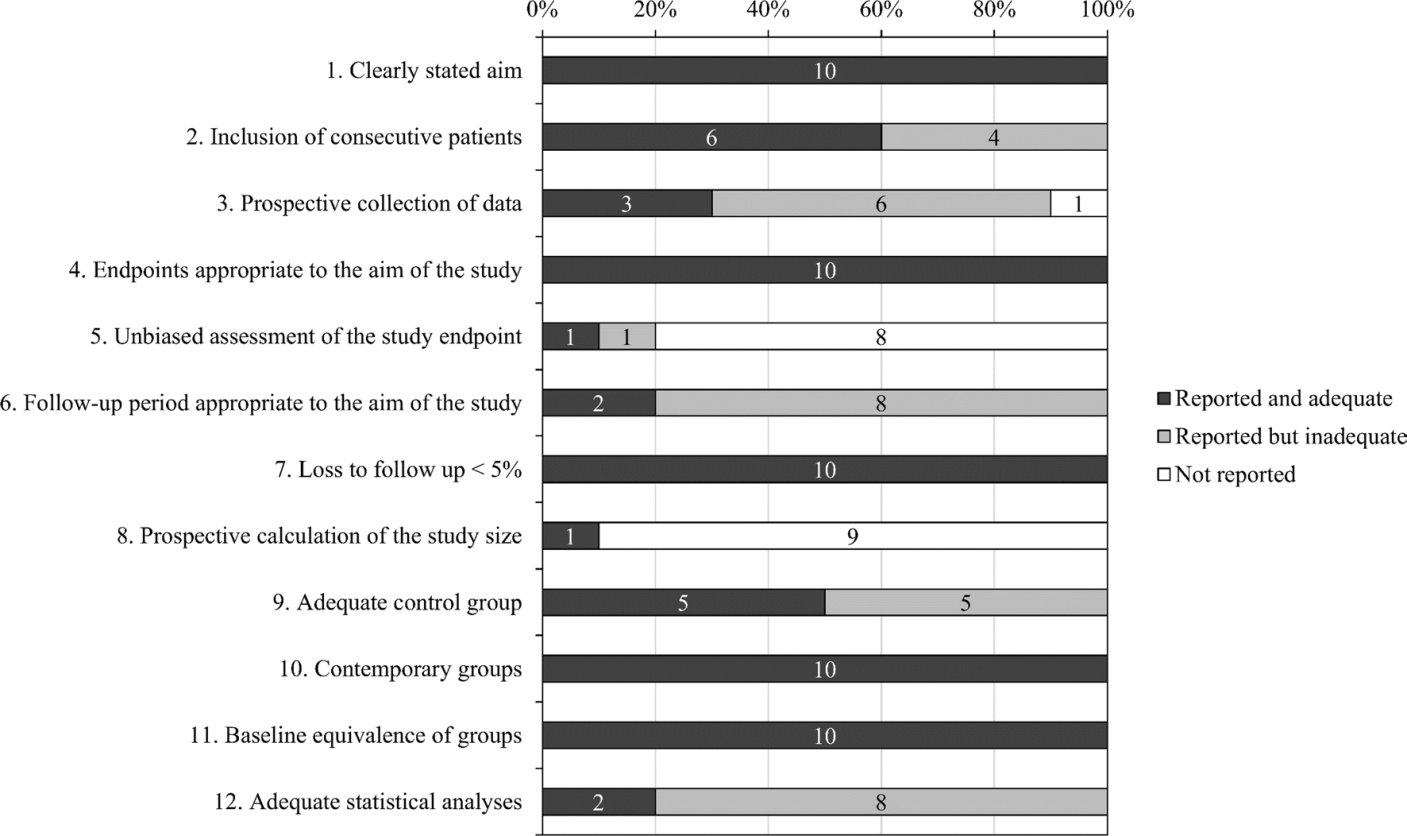
Quality assessment by the Methodological Index for non‐randomized studies (MINORS) for the included studies of current meta-analysis.

A total of 10 studies (Table 1) were controlled trials without randomization because no valid randomized controlled trial that fit within the scope of this study was found. The studies were conducted in the data extraction and quality assessment because one of the included articles had two independent studies^28^. Krippendorff’s alpha of quality assessment was 0.96, indicating good reliability in the process of quality assessment. The evaluation results are presented in Supplementary Table S1. Figure 2 illustrates the quality assessment and represents a risk of bias. The scores of the included 10 studies ranged from 14 to 23. Two studies were assessed to be of fair quality and excellent quality, respectively. Meta-analysis was conducted using the 10 included studies because the quality assessment of all included studies was higher than poor quality. All datasets of included studies in the meta-analysis are shown in Supplementary Table S2.

**Table 1:** Summary of characteristics of studies in current meta-analysis.

Study	Journal	Study design	Sample number	Age (year)	Sample characteristics	Duration and frequency	Activity type	Cognitive measure	Country	Language of article	Quality assessment^a^
Chang (2006) Study I^28^	Master thesis	Non-RCT	22 E	41.6 ± 10.0	Schizophrenia	Twice-weekly during 16 weeks	IP, OP, PR	ACL	Taiwan	Chinese	17/24
			24 C	45.7 ± 8.5							
Chang (2006) Study II^28^	Master thesis	Non-RCT	19 E	42.8 ± 10.8	Schizophrenia	Twice-weekly during 16 weeks	IP, OP, PR	CDT	Taiwan	Chinese	17/24
			20 C	44.5 ± 9.3							
Chen (2008)^29^	Master thesis	Non-RCT	10 E	82.7 ± 5.4	Elderly with chronic disease	Once-weekly during 8 weeks	IP, AC	HTEF-CA	Taiwan	Chinese	18/24
			10 C	81.3 ± 6.5							
Yun and Kim (2009)^32^	Journal	Non-RCT	14 E	78.1	Elderly with dementia	Once-weekly during 8 weeks	IP, AC	MMSE	South Korea	Korean	15/24
			14 C	78.2							
Yun et al. (2010)^33^	Journal	Non-RCT	9 E	76.2	Elderly women	Once-weekly during 18 weeks	IP, AC	MSQ	South Korea	Korean	15/24
			9 C	83.1							
Chung (2014)^30^	Master thesis	Non-RCT	33 E	50.5 ± 7.7	Female with schizophrenia	Once-weekly during 12 weeks	IP, OP, AC, PR	COTE-TB	Taiwan	Chinese	23/24
			30 C	48.8 ± 9.2							
Masuya et al. (2014)^23^	Journal	Non-RCT	9 E	89.0 ± 7.1	Elderly with no dementia	Once-weekly during 6 weeks	IP	MMSE	Japan	English	17/24
			9 C	82.2 ± 6.6							
Park et al. (2016)^31^	Journal	Non-RCT	24 E	79.4 ± 4.8	Elderly women	Twice-weekly during Sept. to Nov	OP	MMSE	South Korea	English	14/24
			26 C	84.5 ± 4.7							
Lee et al. (2017)^24^	Journal	Non-RCT	26 E^b^	80.2 ± 7.0	Elderly with dementia	Once-weekly during 10 weeks	AC, PR	MMSE	South Korea	English	16/24
			9 C	78.7 ± 9.6							
Kenmochi et al. (2019)^17^	Journal	Non-RCT	11 E	55.8 ± 7.5	Schizophrenia with no dementia	Once-weekly during 11 weeks	IP	PANSS-CF	Japan	English	20/24
			12 C	53.0 ± 8.9							

*Non-RCT* non-randomized controlled trial, *E* experimental group, *C* control group, *IP* indoor plant activity, *OP* outdoor plant activity, *AC* arts and craft activities, *PR* other plant-related activities, *ACL* allen cognitive level test, *MMSE* mini-mental state examination, *CDT* clock drawing test, *HTEF-CA* horticultural therapy evaluation form—cognitive ability, *MSQ* mental status questionnaire, *COTE-TB* comprehensive occupational therapy evaluation—task behavior, *PANSS-CF* positive and negative syndrome scale—cognitive factor.

^a^Quality scores derived from Methodological Index for Non‐Randomized Studies (MINORS).

^b^Three experimental groups of Lee et al. (2017) were combined in this study.

### Results of meta-analysis

**Figure 3:**
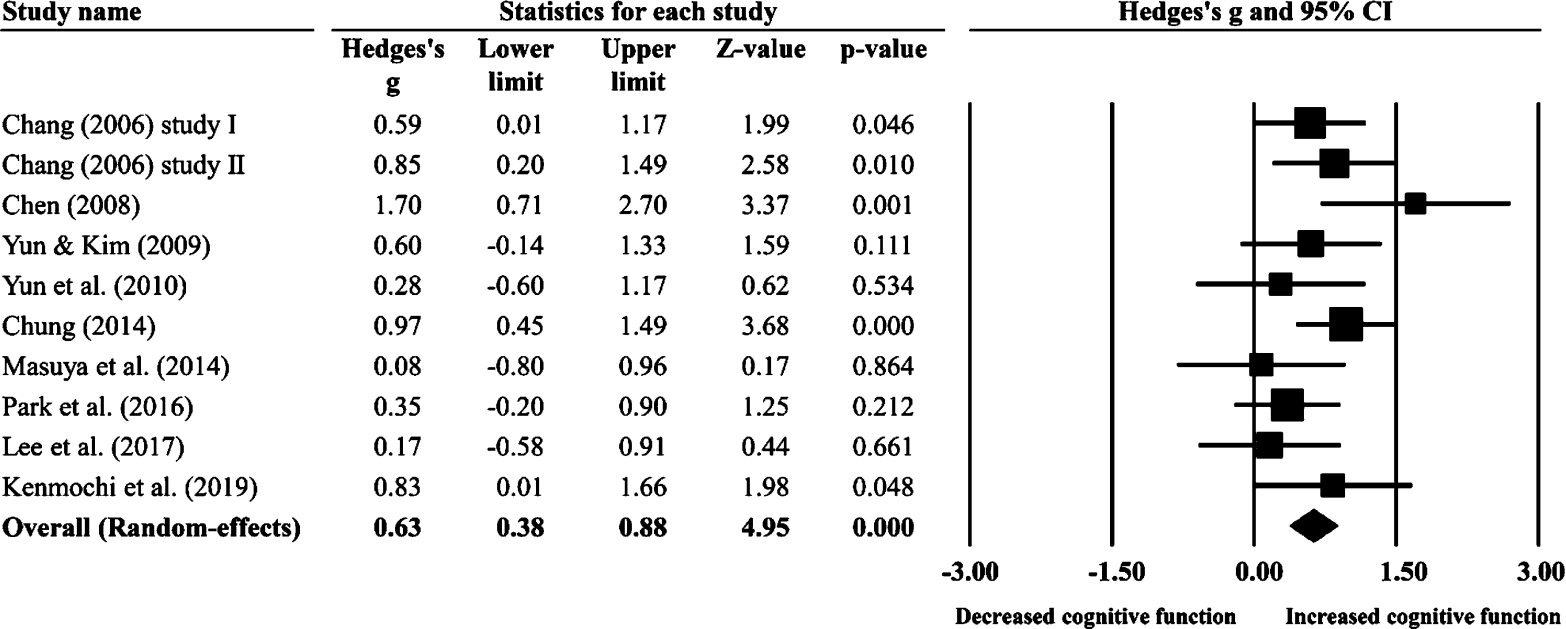
Forest plot: effect sizes (Hedges g) and 95% confidence intervals (95% CI) based on random-effects model in all included studies were evaluated to compare cognitive function between experimental group and control group.

**Figure 4:**
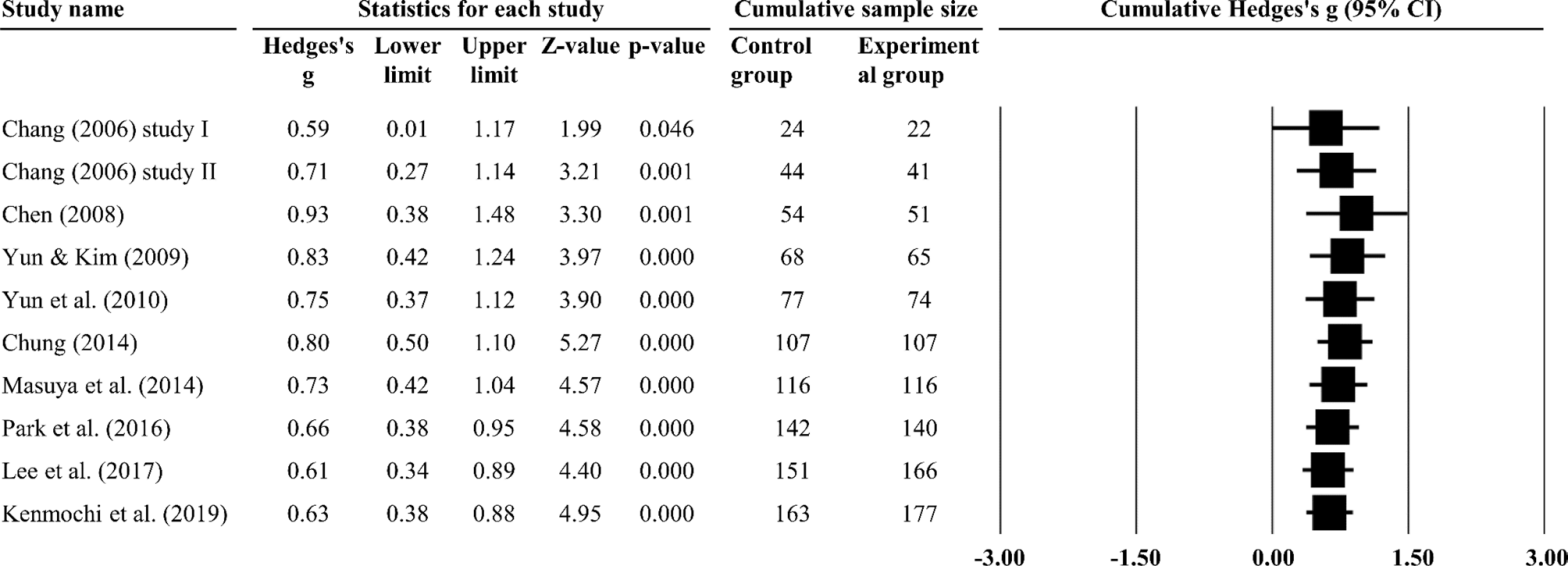
Cumulative meta-analysis with the order of publication year.

**Figure 5:**
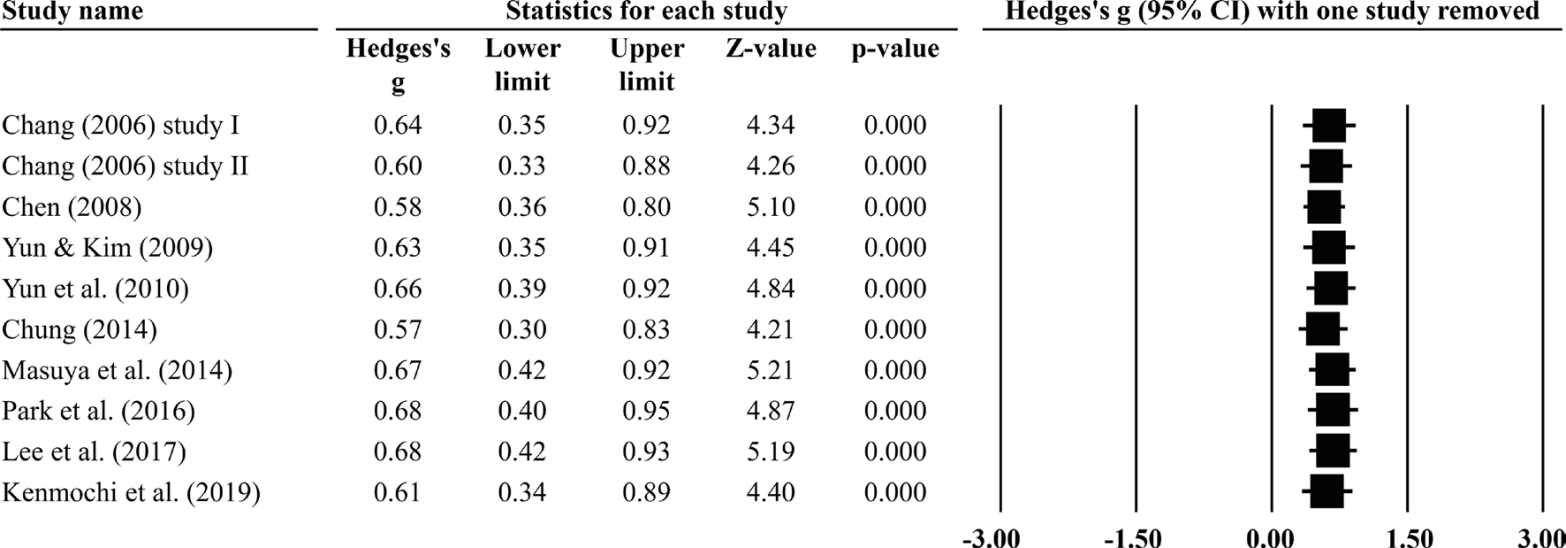
One-study-removed meta-analysis.

This study directly evaluated the overall effect size for all studies because the overall effect size of all studies combined did not present significant heterogeneity (Q = 11.46, df = 9, *p* = 0.245, *I*^2^ = 21.49%, *τ*^2^ = 0.03). The results of the meta-analysis showed a significant improvement in cognitive function (Hedges’ g = 0.63, 95% CI 0.38–0.88, Z = 4.95, *p* < 0.001) (Fig. 3), compared to the control group. Cumulative meta-analysis by study publication year indicated that the effect size tended to stabilize, followed by increasing publication year and number of samples (Fig. 4). There was no evidence of temporal bias. A one-study-removed meta-analysis indicated that removal of any single study still tends to stabilize effect sizes and is still a significant estimate (Fig. 5). This result supports the hypothesis that horticultural therapy programs positively improve cognitive function in the cognitively impaired population when compared with a control group.

### Publication bias

**Figure 6:**
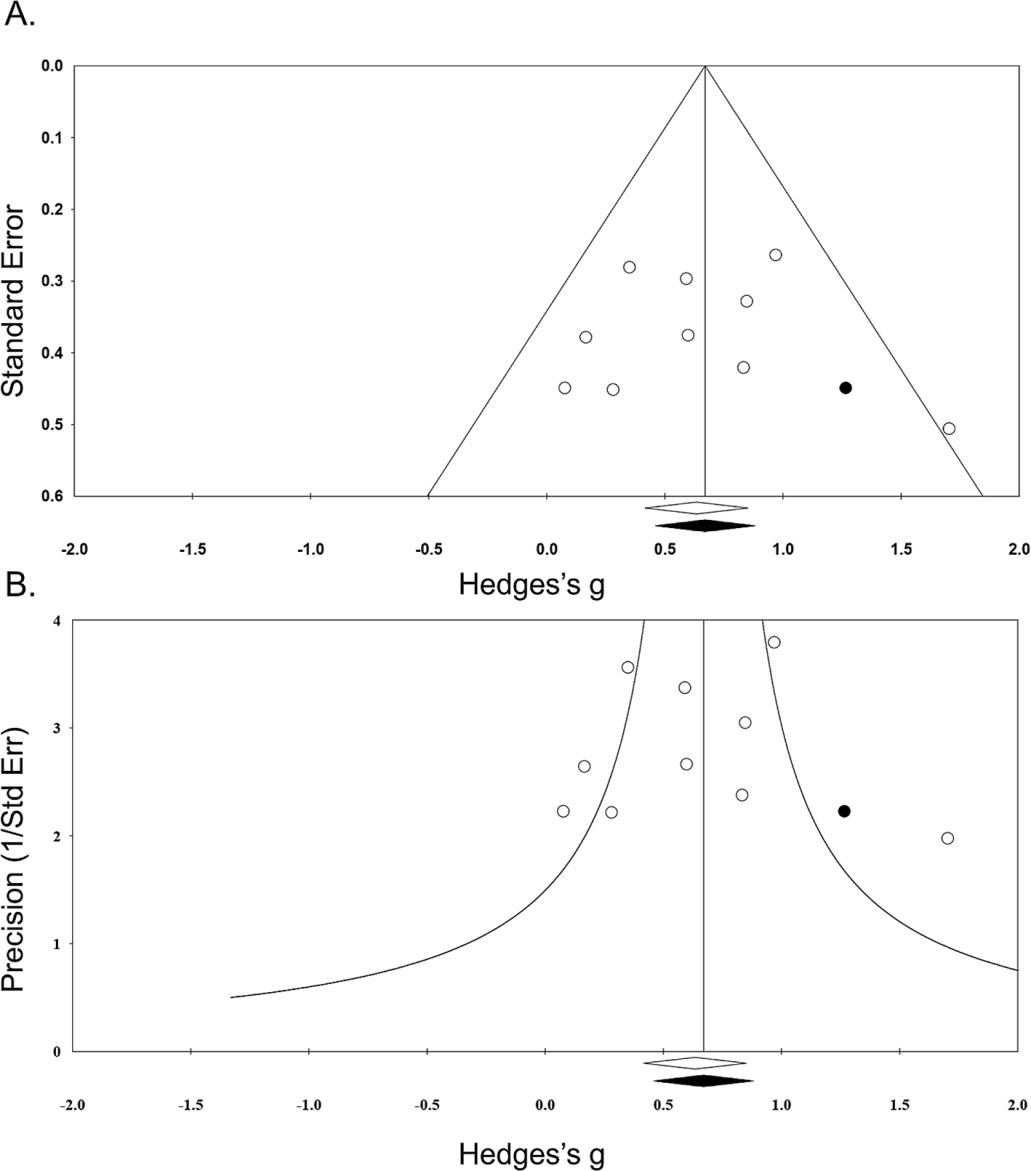
Assessments of publication bias in the meta-analysis. (**A**) Funnel plot with one imputed study of the relationship between the mean effect size and standard error for included study. (**B**) Funnel plot with one imputed study of the relationship between the mean effect size and the precision of included study. The imputed point estimate (Hedges’s g = 0.67; 95% CI 0.42–0.92) was higher than original point estimate (Hedges’s g = 0.63; 95% CI 0.38–0.88).

First, the asymmetrical funnel plot at the bottom of the graph (Fig. 6) indicated that one study with positive point estimate was missing in our meta-analysis, due to potential publication bias. Second, Duval and Tweedie’s trim-and-fill method imputed 1 missing study to the right side of the mean effect and revaluated the point estimate based on a random effects model. The imputed point estimate was 0.67 (95% CI 0.42–0.92), which is higher than the original point estimate of 0.63 (95% CI 0.38–0.88) which is still a significant estimate. Third, the classic fail-safe N (Z = 5.59, *p* < 0.001) indicated that 72 missing null studies were required to change the *p* value to non-significant. Fourth, Orwin’s fail-safe N indicated that we would need to locate 54 studies with mean hedges g of 0.00 to change the effect size down to trivial levels (which we defined as Hedges g < 0.01). Fifth, the Begg and Mazumdar rank correlation was not significant (Kendall’s tau = 0.00; *p* = 1.000). Sixth, Egger’s regression test was not significant (t = 0.00, df = 8, *p* = 0.998). These methods showed that publication bias is insufficient to influence the results of this study.

## Discussion

This meta-analysis of 10 non-randomized controlled trials determined that horticultural therapy significantly improved cognitive function. All 10 included studies were Asian studies because no non-Asian studies were found in search, selection, and identification process for this study. Non-Asian studies were excluded in the process of full-text articles assessed for eligibility due to non-controlled trials. There is no evidence of publication bias affecting the results of this study. The eligible studies included a master thesis, which was also verified to be fair to excellent quality by the process quality. This study selected a random-effects model to test the effect sizes because the nature of included studies was heterogeneous due to different sample characteristics, duration, frequency, and types of horticultural therapy, and cognitive measures. This study directly evaluated the combined effect size of included studies because no statistical heterogeneity was observed between the effect size combined of included studies in the meta-analysis. The effect size was stable and was not affected by the publication year and repeated removal of any single study. This study contributed valuable evidence. Based on this meta-analysis of non-randomized controlled trials: horticultural therapy programs significantly improved cognitive function. The effect size of horticultural therapy was large.

In the process of meta-analysis, we found that the 19 horticultural therapy studies lacked control groups (Fig. 1). Further, this study extracted eight studies with detailed information on the method and results (e.g. mean, S.D., and significance) of the pre-post comparison to provide more information about the pre-post values in the experimental groups. This information is presented in Supplementary Table S3 online, and includes the studies by Park et al.^34^, Lee et al.^35^, Lee and Kim^18^, Hewitt et al.^36^, Lin et al.^37^, Masuya and Ota^38^, Chien and Hsieh^39^, and Yan et al.^40^. Seven studies showed a positive effect direction in the cognitive measure of the experimental group. Although one study had a significant negative effect direction, it explains that the horticultural therapy may have slowed the cognitive decline due to a lower cognitive decline in similar prevalence studies^36^. The all experimental group also have positive effect direction in the included studies of meta-analysis. Overall, the effect direction of experimental group was similar between the included studies and above eight studies without control group.

All included studies presented comparisons of demographic characteristics, socioeconomic, disease, or cognitive function to increase appropriateness of the control group to be compared with the experimental group. This study accessed the inclusion criteria of four included studies^24,31–33^ (e.g. the criteria for inclusion and exclusion, and the reasons for exclusion). Although the four studies reported the inclusion of patients (Supplementary Table S1), the criteria was found to be inadequate. The inadequate information made it difficult to assess the appropriateness of the control group. Six included studies^17,23,28–30^ outlined clear inclusion criteria, such as age, diagnosis of a specific disease by a professional, physical ability, etc. This increased the appropriateness of the control group, enabling comparisons with the experimental group. This study suggested that clear inclusion criteria should be explained in future studies. In addition, while the included studies clearly explained the horticultural therapy program, they rarely explained the activity of control group. This should be also be clearly explained in future studies.

There are certain limitations of this study. The heterogeneity of the sample characteristics, duration, frequency, and types of horticultural therapy, and cognitive measure of included studies may affect the results. However, few related studies have similar parameters. Future study should implement related horticultural therapy studies and meta-analyze the horticultural therapy studies those with similar sample characteristics, activity characteristics, and cognitive measure.

No valid randomized controlled trial that fit within the scope of this study was found. The search strategy and inclusion criteria did not limit randomized controlled trial. Although the meta-analysis of high-quality randomized controlled trials would provide stronger evidence, few horticultural therapy programs executed randomized controlled study designs due to operational difficulties. Recently, an increasing number of studies on horticultural therapy have tried to conduct randomized controlled trials. However, these low-quality randomized controlled trials were not able to contribute to a meaningful meta-analysis^22^.

The field of horticultural therapy is limited by the challenges involved in conducting high-quality randomized controlled trials. First, the process of randomization and blinding should be explained in detail^22^. Although useful results from two relevant protocols were not included in this meta-analysis^41,42^, these protocols clearly describe the process of randomization, blinding, and other steps involved in randomized controlled study designs. Second, promoting the quality of reports is an important step. In this meta-analysis, several studies did not clearly explain the blind evaluation, appropriate follow-up period, study size calculation, and basic descriptive statistics (e.g., means and standard deviations), which are elements that improve the quality of controlled trials.

Due to non-heterogeneity and the small numbers of included studies, further analysis of the duration and frequency of horticultural therapy programs was not carried out. Some studies indicated that spending several hours in gardening activities benefited psychological health^26^. There was a difference in the duration and frequency of horticultural therapy programs among the included studies. One study designed a horticultural therapy program that involved weekly activities for 6 weeks, which did not affect cognitive function^23^. Its small sample size may have affected the results. Another study described a horticultural therapy program of once-weekly activities for 8 weeks, which improved cognitive function^29^. Yet another study obtained opposite results^32^. One study designed a horticultural therapy program of twice-weekly activities for 16 weeks, which affects cognitive function^28^. Due to a lack of heterogeneity among the studies of this meta-analysis, a horticultural therapy program that involved activities for more than once weekly over 8 weeks could be considered as a recommended level to improve cognitive function. Confirming the effect of duration and frequency of horticultural therapy programs on cognitive function is an important issue.

Findings of this meta-analysis provided important evidence that support horticultural therapy for cognitive improvement, which significantly impact on practice and policies. First, medical and healthcare systems should consider horticultural therapy as an important intervention for improving patients’ cognitive function. To date, many countries such as Taiwan, have not integrated horticultural therapy into mainstream medical and healthcare systems due to a lack of high quality evidence from randomized controlled studies, in an era of evidence-based medicine. This study provided higher quality evidence that supported the positive effects of horticultural therapy on cognitive function. More high-quality and more in-depth studies in horticulture are warranted. Population aging induces potential economic and social burdens due to the decline of cognitive function or increased risk of dementia^7,8^. Therefore, governments should consider horticultural therapy or social horticultural activity as important tools to prevent the decline of cognitive function in middle-aged and aging populations.

## Methods

### Search strategy and inclusion criteria

The flow diagram of the Preferred Reporting Items for Systematic Reviews and Meta-Analyses (PRISMA)^43,44^ was used for identification, screening, eligibility, and included the process of this study. In the identification stage, the Google Scholar and Web of Science databases cover the publication of multiple discipline and were used to search for controlled trials that studied the effect of horticultural therapy on cognitive function, by two independent reviewers.

Google Scholar is ideal for searching unpublished and published articles without language restrictions, such as unpublished theses and non-English journals. The search keywords consisted of horticultural therapy, cognitive function, and mini-mental status examination (common measurement tool of cognitive function). The search of Google Scholar and Web of Science were performed by using the search terms “Horticultural therapy” AND “cognitive function” OR “mini-mental status exam” OR “mini-mental status examination”, without language restriction, for articles up to February 2020, without restrictions on publication date. This study also identified additional records from reference lists of identified papers and review articles.

The same independent reviewers implemented screening, eligibility, and inclusion processes. In the screening stage, the same independent reviewers screened the titles and abstracts of articles to determine potentially eligible studies for the topic of horticultural therapy, and excluded irrelevant topics. There were no language limitations. In the stage of eligibility, inclusion criteria were used to exclude ineligible studies: (1) the study design included a control group and an experimental group with pre- and post-test cognitive function; and (2) the experimental group implemented a horticultural therapy program with indoor plant activity, outdoor plant activity, plant-related arts and craft activities, or other plant-related activities. Review articles, study protocols, and abstracts were excluded. Finally, the process of qualitative synthesis was used to determine the feasibility of meta-analysis in each included study and excluded studies within sufficient information for evaluation of effect estimates. The level of agreement between inclusion or exclusion of articles was assessed to ensure reliability, and the process of decision was consistent through Krippendorff’s alpha^45–47^.

### Data extraction and quality assessment

The same independent reviewers separately checked and extracted the data of included articles using a standardized form (Table 1). The standardized form included the study’s author and title, journal name, study design, sample number of experimental group and control group, sample characteristics, activity program, activity type, the measure tool of cognitive function, country, language of article, and quality assessment. In the measure tool of cognitive function, Mini-Mental State Examination (MMSE) is a common cognitive screening tool. Four horticultural therapy studies used MMSE to assess the outcomes of cognitive function^23,24,31,32^. MMSE is also a tool for cognitive outcome measure. A study in the past has indicated a high co-relation with measure of cognitive function, such as Blessed Orientation Memory Concentration^48^. Positive and Negative Syndrome Scale (PANSS) is a measure of psychotic symptoms and includes a factor of cognitive assessment^17^. The study only includes the cognitive factor of PANSS to implement meta-analysis.

Because the final eligible studies did not include randomized controlled trials, the quality of the included non-randomized controlled trials was assessed using the checklist of the Methodological Index for Non-Randomized Studies (MINORS)^49^. The MINORS checklist consists of 12 qualitative items including clearly stated aim, inclusion of consecutive patients, prospective collection of data, endpoints appropriate to the aim of the study, unbiased assessment of the study outcomes, follow-up period appropriate to the aim of the study, loss to follow up less than 5%, prospective calculation of the study size, adequate control group, contemporary groups, baseline equivalence of groups, and adequate statistical analyses^49^. The MINORS checklist used a 3-point scale to assess each quality item, each was scored 0 if not reported, 1 if reported but inadequate, and 2 if reported and adequate^49^. The maximum score of 24 can be categorized into three qualities: 0 to 12 was considered poor quality, 13 to 18 was considered fair quality, and 19 to 24 was considered excellent quality^50^. Quality assessment was evaluated independently by the same reviewers. When inconsistencies occurred between the two independent evaluations, discussion and consensus were used to resolve and reach agreement. The inter-coder reliability was assessed to ensure that the quality assessment was correct and consistent as determined by the Krippendorff’s alpha^45–47^.

### Meta-analysis

To evaluate the effect of horticultural therapy on cognitive function, we calculated the Hedges’ g effect size of the standardized mean difference, which expressed the difference in cognitive function between the experimental group and control group. The value of Hedges’ g effect size of greater than 0 indicated that the cognitive function level was higher in the experimental group than in the control group. The means, standard deviations, and sample sizes (or mean charge in each group, F-value for difference between changes, and sample sizes) of the cognitive function of both the experimental and control groups were used to evaluate the effect sizes for each included study. A random-effects model was used due to the assumption that one common true effect size was not presented in the included studies due to the diversity of horticultural therapy programs (e.g., different studies, different activities, and periods), cognitive measures (e.g., Mini-Mental State Examination, Allen Cognitive Level Test, etc.), and sample characteristics (e.g., age and diseases) as well as to generalize findings beyond the included studies, by assuming that the selected studies are random samples from a larger population^51^.

Heterogeneity between studies was examined using the Q statistic test, *I*^2^ statistic, and τ^2^^25^. In sensitivity analysis, a cumulative meta-analysis of the study publication year was used to check the potential time of publication bias^52^. A one-study-removed meta-analysis was also used to determine if the results of this study were sensitive to the repeated exclusion of a single included study. We assessed potential publication bias using funnel plots^25^, Duval and Tweedie’s trim-and-fill^25,53^, classic (Rosenthals) fail-safe N^25,54^, Orwin’s fail-safe N^25^, Begg and Mazumdar rank correlation^25,55^, and Eggers regression test^25,56^. Comprehensive Meta-Analysis Version 3^57^ was used to conduct the meta-analysis.

## Supplementary information

Supplementary Information

## Data Availability

Data that supports the findings of this study are included in Supplementary Tables.
