# Supplementary material for: Meta-analysis of controlled trials testing horticultural therapy for the improvement of cognitive function
Source: Sci Rep. 2020 Sep 3;10:14637. doi: 10.1038/s41598-020-71621-7 (PMC7471303; doi:10.1038/s41598-020-71621-7)
Supplement: Supplementary file 1 — Supplementary Information [file 41598_2020_71621_MOESM1_ESM.pdf]

Meta-analysis of controlled trials testing horticultural therapy for the improvement of cognitive function

Hung-Ming Tu[1*], Pei-Yu Chiu[1]

[1]Department of Horticulture, National Chung Hsing University, Taichung, 40227, Taiwan
[*]hmtu@dragon.nchu.edu.tw

Supplementary Table S1 Quality assessment by the Methodological Index for Non-Randomized Studies for the included studies in current meta-analysis

Study	1 Clearly stated aim	2 Inclusion of consecutive patients	3 Prospective collection of data	4 Endpoints appropriate to the aim of the study	5 Unbiased assessment of the study endpoint	6 Follow-up period appropriate to the aim of the study	7 Loss to follow up < 5%	8 Prospective calculation of the study size	9 Adequate control group	10 Contemporary groups	11 Baseline equivalence of groups	12 Adequate statistical analyses	Score
Chang (2006) Study I [28]	2	2	1	2	0	1	2	0	2	2	2	1	17/24
Chang (2006) Study II [28]	2	2	1	2	0	1	2	0	2	2	2	1	17/24
Chen (2008) [29]	2	2	1	2	0	1	2	0	2	2	2	2	18/24
Yun & Kim (2009) [32]	2	1	1	2	0	1	2	0	1	2	2	1	15/24
Yun et al. (2010) [33]	2	1	1	2	0	1	2	0	1	2	2	1	15/24
Chung (2014) [30]	2	2	2	2	2	2	2	2	2	2	2	1	23/24
Masuya et al. (2014) [23]	2	2	2	2	0	1	2	0	1	2	2	1	17/24
Park et al. (2016) [31]	2	1	0	2	0	1	2	0	1	2	2	1	14/24
Lee et al. (2017) [24]	2	1	1	2	0	1	2	0	1	2	2	2	16/24
Kenmochi et al. (2019) [17]	2	2	2	2	1	2	2	0	2	2	2	1	20/24

NOTE. Index is as follows: 0, not reported; 1, reported but inadequate; and 2, reported and adequate.

Supplementary Table S2 All datasets of included studies in the meta-analysis.

Study	Data format	Control group						Experimental group						F for difference
		Sample size	Pre-mean	Pre-S.D.	Post-mean	Post-S.D.	Mean change	Sample size	Pre-mean	Pre-S.D.	Post-mean	Post-S.D.	Mean change	
Chang (2006) Study I [28]	Mean, S.D. in each group	24	3.910	0.920	3.860	0.790		22	4.590	1.010	5.090	0.920		
Chang (2006) Study II [28]	Mean, S.D. in each group	20	22.900	5.710	21.650	5.930		19	20.890	7.470	25.000	4.910		
Chen (2008) [29]	Mean, S.D. in each group	10	2.620	0.662	2.860	0.550		10	2.880	0.784	4.400	0.837		
Yun & Kim (2009) [32]	Mean, S.D. in each group	14	10.570	7.510	9.070	7.480		14	10.430	6.110	13.290	7.040		
Yun et al. (2010) [33]	Mean, S.D. in each group	9	6.670	2.400	6.560	2.510		9	6.110	3.060	6.780	2.490		
Chung (2014) [30]	Mean, S.D. in each group	30	11.670	2.450	12.500	3.820		33	11.030	2.850	15.270	4.020		
Masuya et al. (2014) [23]	Mean, S.D. in each group	9	24.300	4.800	24.200	5.100		9	23.100	4.800	23.400	5.100		
Park et al. (2016) [31]	Mean, S.D. in each group	26	22.100	3.700	21.800	3.500		24	22.600	4.200	23.600	2.800		
Lee et al. (2017) [24]	Mean, S.D. in each group	9	19.400	3.700	19.500	4.100		26	16.115	4.200	16.923	4.300		
Kenmochi et al. (2019) [17]	Change, F for difference in Change	12					1.080	11					-2.000	4.290

Supplementary Table S3 Pre-post values of the horticultural therapy study without control groups

Study	Cognitive measure	Experimental group							Significant test in original study	Effect direction
		Sample size	Pre-mean	Pre-S.D.	Pre-range	Post-mean	Post-S.D.	Post-range		
Park et al. (2003) [34]	MMSE	7	22.000	5.900		23.430	6.700		No significant (p > 0.05)	Positive
Lee et al. (2007) [35]	EHA	9	3.100	1.290		4.100	0.870		Significant (p < 0.01)	Positive
Lee & Kim (2008) [36]	HDS-R	23	13.700	3.770		17.480	4.100		Significant (p < 0.001)	Positive
Hewitt et al. (2013) [37]	MMSE	9	17.000		8-28	15.870		0-26	Significant (p < 0.05)	Negative
Lin et al. (2014) [38]	MMSE	8	15.380	3.852		17.630	4.104		Significant (p < 0.05)	Positive
Masuya & Ota (2014) [39]	MMSE	11	17.000		11-22	19.000		14-23	Significant (p < 0.01)	Positive
Chien & Hsieh (2015) [40]	EHA	10	3.380	0.310		3.950	0.210		Significant (p < 0.01)	Positive
Yan et al. (2016) [41]	EHA	11	35.500	2.780		51.170	2.760		Significant (p < 0.01)	Positive

NOTE. EHA, Evaluation of horticultural activity from past studies; HDS-R, Revised Hasegawa's Dementia Scale; MMSE, Mini-Mental State Examination
